# The Condition of the Pupil during the Administration of Chloroform

**Published:** 1874-11-15

**Authors:** 


### THE CONDITION OF THE PUPIL DURING THE ADMINISTRATION OF CHLOROFORM.

*Translated from La France Medicale, by F. J. Huse, M.D.*

IN none of our books does there appear any sign to guide us in the administration of chloroform; neither is any symptom described from which we may learn the probable condition of sensibility, except such as may be obtained from careful watching of the movements and moanings of the patient. These moanings and movements, however, must of necessity cease in the course of operations of long duration, as for example, ovariotomy and hysterotomy.

In carefully examining all the details in connection with the administration of chloroform, it has seemed to us that there exists a certain connection between the condition of the pupils and the degree of the patient's anæsthesia. We have remarked, also, that the efforts to vomit, which are so frequently met with in surgical anæsthesia, produce modifications, both in the condition, pupil, and in the general sensibility. Notwithstanding the conflicting notions that have prevailed regarding the contraction and dilatation of the iris in anæsthesia, ideas which we do not here propose to discuss, we have continued our investigations and have reached certain results, which, if confirmed by other observers, will probably prove not entirely devoid of a certain degree of utility in every-day practice.

Aided by our friend and former colleague, Dr. Coyne, we reproduced, by experimentation upon animals, in the laboratory directed by Prof. Vulpian, the very same results that we had observed in the human species. The detailed account of these observations we purpose giving at some future time, at present restricting ourselves to the publication of a brief recapitulation of our researches.

As advised by the majority of hospital surgeons, we administered chloroform gradually, in order to accustom the patient to its use, giving it upon a compress or handkerchief, and allowing the air to permeate freely. We have often witnessed the rapid supervention of death in dogs whose muzzles were covered with a rubber sack that contained a sponge saturated with chloroform, and presented too narrow an opening at the opposite extremity. With this compress, which can be speedily removed whenever any difficulty of respiration may appear, anæsthesia is more gradual (ten to thirty minutes), but accidents seem to be less frequent than if the patient is overwhelmed at the beginning by large quantities of the anæsthetic.

Regarding the condition of the pupil: When the period of excitation approaches, the pupil, which up to that time was mobile, becomes insensible to the light and *dilates*. The patient still retains cutaneous sensibility, shrinking and crying out when pinched. This period of excitation is sometimes very short—it may even escape notice.

After this stage of excitement, the pupil, still remaining insensible to the light, slowly *contracts*, and this contraction becomes more and more evident. If the patient is now pinched, or if the operation is commenced, the pupil relaxes, and sometimes reaches the maximum dilatation. At the same time the subject draws up his extremities, struggles, groans, or cries out.

If, on the contrary, the pupils being contracted, the administration of the chloroform is patiently continued for five or six minutes, the contraction persists and becomes somewhat more strongly marked. If the subject now be pinched there is no longer any dilatation of the pupils, nor any movement, nor any groaning; the operation may proceed, the patient is insensible. This state of complete anæsthesia often coincides with the onset of snoring.

During the operation, especially if it is of long duration, it is necessary to endeavor to preserve this occlusion of the pupils by continuing the administration of chloroform, since with the dilatation of the pupils, whenever allowed to become too great, there is also a re-appearance of sensibility—marked by movements and groans, then the awakening. If, however, the pupils remain well controlled, the patient continues quiet and insensible.

It is very noticeable that the patient's insensibility is exhausted progressively, and in the same ratio as the step in the operation would be painful to him if he was not under the influence of chloroform. Incisions of the skin are especially liable to produce dilatation of the pupil and awakening, if the impression of the anæsthetic is not very profound.

In an instance where the patient was addicted to the use of alcoholic stimulus, and where, after a very long period of excitement, we could only obtain an incomplete anæsthesia, the contraction of the pupils was much less than in other cases. When, at the termination of the operation, he was allowed to return gradually to consciousness, the pupils slowly dilated, then the patient awoke and the pupils became sensitive to light.

These same phenomena were observed during our experiments upon animals. In administering chloroform to dogs it was observed that the dilatation of the pupils coincided with the stage of excitement, and that the contraction of the pupil was immediately succeeded by insensibility. Likewise, if after one or two minutes had passed a dog was forcibly punched, or if his paw was trodden upon, his pupils dilated immediately, he moved and whined. Continuing the chloroform, however, and waiting until his pupils had been contracted for from five to ten minutes, the crushing of his paw was not perceived, but a very strong current produced, after a lapse of one or two minutes, movements and outcries which coincided with a rapid and almost instantaneous dilatation of his pupils. Ceasing the electrical excitation and continuing the effects of the chloroform further, the pupils were greatly contracted, and tickling the velum of the palate aroused no motions, but when the sciatic nerve was laid bare and pinched the animal drew up his paw and his pupils dilated to a slight extent.

Again, pushing the anæsthesia to a still greater degree, the sciatic nerve could be tied, and after being severed, its central extremity could be excited without producing any movement or any dilatation of the pupils. Moreover, the great sympathetic and pneumogastric nerves could be exposed in the neck and subjected to the action of strong currents of electricity without awakening the dog. During all these operations the pupil remained strongly contracted. The animal awoke of his own accord an hour later, and survived, without presenting any accident attributable to the anæsthetic.

There exists, therefore, in the anæsthesia produced by chloroform for surgical purposes, a relation between the complete insensibility of the patient and the contraction of the pupil; between the return to sensibility and the dilatation of this organ.

Moreover, although we believe that this fact has not yet been published, it is the custom in the laboratory of Prof. Vulpian to use the state of the pupil as an index of the completeness of the anæsthesia, after intravenous injections of chloral. If, upon laying bare the sciatic nerve and pinching it, the pupil proves to be immobile, its insensibility has always, in the experience of M. Carville, been a reliable indication.

In concluding this topic we should mention that in the case of two dogs, where syncope and death followed the administration of large quantities of chloroform, there succeeded almost instantaneously to the contraction of the pupils a complete dilatation.

Quite often during surgical anæsthesia there are attacks of vomiting. These vomitings, accompanied by strugglings, have consequences which are worthy of study, and which vary somewhat, according to the stage of anæsthesia in which they make their appearance. The following instances have fallen under our observation:

In a case where the administration of chloroform had only been made for a brief period of time, the patient having lost his consciousness, the pupils being slightly contracted and anæsthesia commenced, an attack of vomiting came on. The face, which had been pale, flushed up, the conjunctiva became injected, the pupils relaxed and remained dilated, while at the same time sensibility entirely re-appeared; consciousness also returned to the patient and he responded to questions.

In another instance, anæsthesia being more complete, and the pupils having been contracted for five minutes, the operation was begun as the patient was insensible. Just then, however, vomiting took place; the face became suffused, the pupils dilated, and sensibility reappeared, though without the return of the patient to consciousness. The administration of chloroform was continued and the operation was delayed from seven to eight minutes.

On other occasions it was in the course of the operation and while anæsthesia was quite profound, that vomiting appeared. In such cases there was, likewise, congestion of the face, dilatation of the pupils, and a return to sensibility, marked by some movement of the patient. However, by increasing the quantity of the anæsthetic the contraction of the pupils and insensibility were speedily restored.

Again, we have frequently seen vomiting come on after the termination of the operation, and when the administration of chloroform had been discontinued for some time; anæsthesia being then less profound, the pupils became rapidly dilated and the patients were speedily and completely aroused.

We have endeavored to reproduce these same phenomena in dogs which had been anæsthetized with chloroform, by injecting apomorphine subcutaneously. We have obtained results that were similar but less strongly marked: similar in so far as related to the dilatation of the pupils, the return of sensibility and the transitory awakening; less strongly marked, since it is difficult to ascertain the efforts at vomiting in dogs submitted to the influence of chloroform, and when they survive these efforts are less numerous than in man.

It has seemed interesting to us to report these items at this time, when Dr. C. J. Campbell, in a memoir distinguished by more than one mark of honor, has suggested the phenomenon of the exertion during delivery, and the resulting cerebral congestion, as the cause of the almost absolute harmlessness of anæsthesia in cases of childbirth.

In reviewing our observations and experiments we believe that we are warranted in drawing the following conclusions:

If we publish them to-day, while purposing to continue this line of investigation, we are only following the counsels of our preceptors, who have urged us to do so on account of the utility which the subject possesses, and the results which we have obtained. If, moreover, we encounter exceptions to these rules, we bind ourselves to make them known.

I. There exists in the surgical anæsthesia produced by chloroform, a constant relation between the condition of the pupil and the stage of anæsthesia.

II. During the period of excitement the pupil is dilated.

III. This period passed the pupil contracts, its occlusion, when well marked and lasting for several minutes, being generally accompanied by complete anæsthesia.

IV. Dilatation of the pupil occurring in the course of the operation generally indicates that the anæsthesia is less profound, and that a return of sensibility is to be apprehended.

V. The condition of the pupil may therefore serve as a guide in the administration of chloroform.

VI. During operations of long duration, if it is desirable that the patient should be entirely insensible and motionless, it is necessary to direct the anæsthesia in such a manner that the pupils remain constantly contracted.

VII. Finally, the effort of vomiting can produce dilatation of the pupils, cause insensibility to disappear, and awaken the patient ; it overcomes, in part, the effects of anæsthesia.

